# *N*-(Cyano­meth­yl)benzamide

**DOI:** 10.1107/S1600536810003557

**Published:** 2010-02-06

**Authors:** Younas Aouine, Anouar Alami, Abdelilah El Hallaoui, Abdelrhani Elachqar, Hafid Zouihri

**Affiliations:** a Laboratoire de Chimie Organique, Faculté des Sciences Dhar el Mahraz, Université Sidi Mohammed Ben Abdellah, Fès, Morocco; b Centre National pour la Recherche Scientifique et Technique, Division UATRS, Rabat, Morocco

## Abstract

In the structure of the title compound, C_9_H_8_N_2_O, the amide group is twisted by a dihedral angle of 21.86 (7)° with respect to the benzene ring, while the planes of the benzene ring and cyano­methyl group form a dihedral angle of 53.13 (11)°. In the crystal structure, mol­ecules are linked *via* N—H⋯O hydrogen bonds, forming a chain running parallel to the *a* axis.

## Related literature

For the biological activity and medicinal properties of tetra­zole derivatives, see: Smissman *et al.* (1976 ▶); McGuire *et al.* (1990 ▶); Lunn *et al.* (1992 ▶); Itoh *et al.* (1995 ▶); Upadhayaya *et al.* (2004 ▶); Wu *et al.* (2008 ▶); Rostom *et al.* (2009 ▶); Burger (1991 ▶); Singh *et al.* (1980 ▶). For the synthetic procedure, see: Adams & Langley (1941*a* ▶,*b* ▶). 
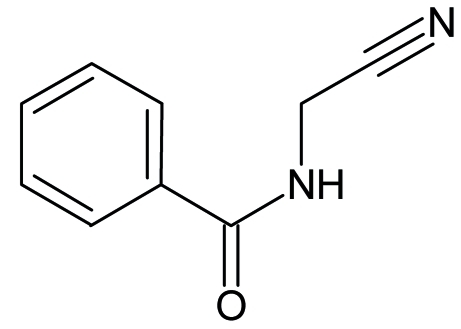


## Experimental

### 

#### Crystal data

C_9_H_8_N_2_O
*M* *_r_* = 160.17
Orthorhombic, 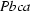

*a* = 9.8623 (5) Å
*b* = 8.0576 (4) Å
*c* = 20.9268 (9) Å
*V* = 1662.98 (14) Å^3^
*Z* = 8
Mo *K*α radiation
μ = 0.09 mm^−1^
*T* = 296 K
0.33 × 0.28 × 0.22 mm

#### Data collection

Bruker X8 APEXII CCD area-detector diffractometer
11132 measured reflections
1920 independent reflections
1433 reflections with *I* > 2σ(*I*)
*R*_int_ = 0.027

#### Refinement

*R*[*F* ^2^ > 2σ(*F* ^2^)] = 0.038
*wR*(*F* ^2^) = 0.109
*S* = 1.02
1920 reflections
141 parameters
H atoms treated by a mixture of independent and constrained refinement
Δρ_max_ = 0.14 e Å^−3^
Δρ_min_ = −0.18 e Å^−3^

### 

Data collection: *APEX2* (Bruker, 2005 ▶); cell refinement: *SAINT* (Bruker, 2005 ▶); data reduction: *SAINT*; program(s) used to solve structure: *SHELXS97* (Sheldrick, 2008 ▶); program(s) used to refine structure: *SHELXL97* (Sheldrick, 2008 ▶); molecular graphics: *PLATON* (Spek, 2009 ▶) and *ORTEP-3 for Windows* (Farrugia, 1997 ▶); software used to prepare material for publication: *publCIF* (Westrip, 2010 ▶).

## Supplementary Material

Crystal structure: contains datablocks I, global. DOI: 10.1107/S1600536810003557/dn2533sup1.cif

Structure factors: contains datablocks I. DOI: 10.1107/S1600536810003557/dn2533Isup2.hkl

Additional supplementary materials:  crystallographic information; 3D view; checkCIF report

## Figures and Tables

**Table 1:** Hydrogen-bond geometry (Å, °)

*D*—H⋯*A*	*D*—H	H⋯*A*	*D*⋯*A*	*D*—H⋯*A*
N1—H6⋯O1^i^	0.819 (17)	2.021 (18)	2.8313 (14)	169

Symmetry code: (i) 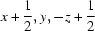.

**Fig. 1.**
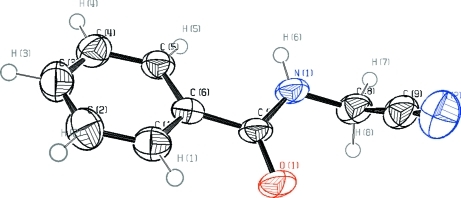
: Molecular structure of the title compound with the atom-labelling scheme. Displacement ellipsoids are drawn at the 50% probability level. H atoms are represented as small spheres of arbitrary radii.

**Fig. 2.**
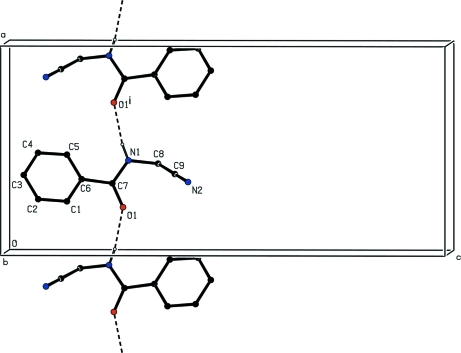
: Partial packing view showing the chain generated by N—H···O hydrogen bonds shown as dashed lines. H atoms not involved in hydrogen bonding have been omitted for clarity.[Symmetry code: (i) x+1/2,y,-z+1/2].

## supplementary crystallographic information

### Comment

Tetrazoles derivatives are an important class of compounds, which can be used in the fields of bioorganic and medicinal chemistry as antibacterials, anti-cancer, heart disease, neurodegenerative disease, and antifungal activity (Smissman *et al.*,1976; McGuire *et al.*, 1990; Lunn *et al.*, 1992; Itoh *et al.*, 1995; Upadhayaya *et al.*, 2004; Wu *et al.*, 2008; Rostom *et al.*, 2009.

The tetrazole moiety has long been established as a bioisostere of a carboxyl unit (Burger, 1991). A major advantage of tetrazoles over carboxylic acids is that they are resistant to many biological metabolic degradation pathways (Singh *et al.*, 1980).

With the aim of developing new tetrazolic derived, an analog isosteric of the glycine, we have prepared *N*-(cyanomethyl)benzamide, a key intermediate, starting from aminoacetonitrile hydrogen sulphate.

In the title compound, the amide group is rotated by 21,86° out of the plane of the benzene ring (Fig. 1). The crystal packing is stabilized by N—H···O hydrogen bonds (Table 1) to form infinite chains parallel to the a axis (Fig. 2).

### Experimental

Aminoacetonitrile hydrogen sulphate was prepared in two steps from technical formaldehyde (Adams & Langley, 1941a,b).

To a solution of 10 mmol s of aminoacetonitrile hydrogen sulfate in 10 ml of methylene chloride, triethylamine was added until neutral pH at cold temperature (0 <T< 5°C ), then 11 mmol s of Benzoyl chloride was added at the same temperature. The mixture was stirred at 0°C for 1 hour. The whole is taken to room temperature and left under magnetic agitation during 16 hours. After reaction, the mixture was washed 3 times with a solution of citric acid 15%; then, the organic solution is dried over sodium sulphate and evaporated under reduced pressure. The residue was crystallized from a mixture ether/hexane (1:1) to give white solid in 82% yield. m.p.: 138- 140°C .

The structure of the product was established on the basis of NMR spectroscopy (^1^H, ^13^C ), MS data and elemental analysis.

Single crystals of the title compound were obtained from an ethanolic solution and used for X-ray diffraction studies at room temperature.

### Refinement

All H atoms were located in a difference map and refined without any distance restraints.

### Crystal data

**Table:**

C_9_H_8_N_2_O	*D*_x_ = 1.280 Mg m^−^^3^
*M**_r_* = 160.17	Melting point: 413 K
Orthorhombic, *P**b**c**a*	Mo *K*α radiation, λ = 0.71073 Å
Hall symbol: -P 2ac 2ab	Cell parameters from 3689 reflections
*a* = 9.8623 (5) Å	θ = 2.5–27.2°
*b* = 8.0576 (4) Å	µ = 0.09 mm^−^^1^
*c* = 20.9268 (9) Å	*T* = 296 K
*V* = 1662.98 (14) Å^3^	Block, colourless
*Z* = 8	0.33 × 0.28 × 0.22 mm
*F*(000) = 672	

### Data collection

**Table:**

Bruker X8 APEXII CCD area-detector diffractometer	1433 reflections with *I* > 2σ(*I*)
Radiation source: fine-focus sealed tube	*R*_int_ = 0.027
graphite	θ_max_ = 27.6°, θ_min_ = 2.8°
φ and ω scans	*h* = −12→12
11132 measured reflections	*k* = −10→10
1920 independent reflections	*l* = −26→27

### Refinement

**Table:**

Refinement on *F*^2^	Primary atom site location: structure-invariant direct methods
Least-squares matrix: full	Secondary atom site location: difference Fourier map
*R*[*F*^2^ > 2σ(*F*^2^)] = 0.038	Hydrogen site location: inferred from neighbouring sites
*wR*(*F*^2^) = 0.109	H atoms treated by a mixture of independent and constrained refinement
*S* = 1.02	*w* = 1/[σ^2^(*F*_o_^2^) + (0.0541*P*)^2^ + 0.2439*P*] where *P* = (*F*_o_^2^ + 2*F*_c_^2^)/3
1920 reflections	(Δ/σ)_max_ < 0.001
141 parameters	Δρ_max_ = 0.14 e Å^−^^3^
0 restraints	Δρ_min_ = −0.18 e Å^−^^3^

### Special details

**Table:**

Geometry. All esds (except the esd in the dihedral angle between two l.s. planes) are estimated using the full covariance matrix. The cell esds are taken into account individually in the estimation of esds in distances, angles and torsion angles; correlations between esds in cell parameters are only used when they are defined by crystal symmetry. An approximate (isotropic) treatment of cell esds is used for estimating esds involving l.s. planes.
Refinement. Refinement of *F*^2^ against ALL reflections. The weighted *R*-factor wR and goodness of fit *S* are based on *F*^2^, conventional *R*-factors *R* are based on *F*, with *F* set to zero for negative *F*^2^. The threshold expression of *F*^2^ > σ(*F*^2^) is used only for calculating *R*-factors(gt) etc. and is not relevant to the choice of reflections for refinement. *R*-factors based on *F*^2^ are statistically about twice as large as those based on *F*, and *R*- factors based on ALL data will be even larger.

### Fractional atomic coordinates and isotropic or equivalent isotropic displacement parameters (Å^2^)

**Table:**

	*x*	*y*	*z*	*U*_iso_*/*U*_eq_	
C1	0.24170 (14)	0.39220 (16)	0.13675 (7)	0.0488 (3)	
C2	0.25299 (17)	0.43033 (18)	0.07283 (7)	0.0591 (4)	
C3	0.36704 (17)	0.38418 (18)	0.03941 (7)	0.0592 (4)	
C4	0.47054 (16)	0.30131 (19)	0.06980 (7)	0.0552 (4)	
C5	0.46079 (13)	0.26308 (16)	0.13418 (6)	0.0448 (3)	
C6	0.34534 (11)	0.30793 (14)	0.16810 (6)	0.0387 (3)	
C7	0.32434 (11)	0.26377 (15)	0.23629 (6)	0.0415 (3)	
C8	0.41628 (14)	0.17663 (17)	0.33761 (6)	0.0498 (3)	
C9	0.37055 (14)	0.31427 (19)	0.37822 (6)	0.0529 (3)	
O1	0.21031 (8)	0.25889 (15)	0.26031 (5)	0.0645 (3)	
N1	0.43271 (11)	0.22652 (14)	0.27184 (5)	0.0456 (3)	
N2	0.33580 (17)	0.4211 (2)	0.40988 (7)	0.0792 (4)	
H1	0.1646 (16)	0.4224 (18)	0.1609 (7)	0.064 (4)*	
H2	0.1826 (17)	0.489 (2)	0.0525 (8)	0.075 (5)*	
H3	0.3723 (16)	0.410 (2)	−0.0064 (8)	0.073 (5)*	
H4	0.5496 (17)	0.267 (2)	0.0484 (8)	0.067 (5)*	
H5	0.5302 (16)	0.2004 (18)	0.1546 (7)	0.054 (4)*	
H6	0.5098 (18)	0.2419 (17)	0.2585 (7)	0.053 (4)*	
H7	0.5037 (17)	0.1326 (19)	0.3548 (8)	0.065 (4)*	
H8	0.3480 (15)	0.0870 (17)	0.3409 (7)	0.057 (4)*	

### Atomic displacement parameters (Å^2^)

**Table:**

	*U*^11^	*U*^22^	*U*^33^	*U*^12^	*U*^13^	*U*^23^
C1	0.0411 (7)	0.0482 (7)	0.0571 (8)	0.0051 (6)	−0.0007 (6)	0.0009 (6)
C2	0.0638 (9)	0.0554 (8)	0.0581 (8)	0.0075 (7)	−0.0087 (7)	0.0066 (7)
C3	0.0733 (11)	0.0587 (8)	0.0457 (7)	−0.0087 (8)	−0.0014 (7)	0.0005 (6)
C4	0.0508 (8)	0.0631 (8)	0.0517 (7)	−0.0064 (7)	0.0110 (6)	−0.0100 (6)
C5	0.0333 (6)	0.0512 (7)	0.0500 (7)	−0.0016 (5)	0.0015 (5)	−0.0043 (5)
C6	0.0290 (5)	0.0400 (5)	0.0470 (6)	−0.0038 (5)	−0.0013 (5)	−0.0023 (5)
C7	0.0240 (5)	0.0503 (6)	0.0502 (7)	−0.0016 (5)	0.0027 (5)	0.0016 (5)
C8	0.0373 (7)	0.0564 (8)	0.0558 (7)	0.0041 (6)	0.0010 (6)	0.0149 (6)
C9	0.0435 (7)	0.0665 (8)	0.0487 (7)	0.0021 (6)	0.0020 (6)	0.0156 (7)
O1	0.0224 (4)	0.1126 (9)	0.0586 (6)	−0.0004 (5)	0.0037 (4)	0.0142 (5)
N1	0.0236 (5)	0.0632 (7)	0.0500 (6)	−0.0002 (5)	0.0027 (4)	0.0093 (5)
N2	0.0867 (11)	0.0836 (9)	0.0675 (8)	0.0108 (8)	0.0100 (8)	−0.0003 (7)

### Geometric parameters (Å, °)

**Table:**

C1—C2	1.377 (2)	C5—H5	0.952 (16)
C1—C6	1.3915 (17)	C6—C7	1.4852 (17)
C1—H1	0.945 (16)	C7—O1	1.2324 (13)
C2—C3	1.376 (2)	C7—N1	1.3364 (15)
C2—H2	0.940 (18)	C8—N1	1.4430 (17)
C3—H3	0.984 (16)	C8—C9	1.468 (2)
C4—C3	1.376 (2)	C8—H8	0.990 (15)
C4—H4	0.940 (17)	C8—H7	0.999 (17)
C5—C4	1.3854 (19)	C9—N2	1.1389 (19)
C5—C6	1.3896 (17)	N1—H6	0.820 (17)
			
C5—C6—C1	119.21 (12)	N1—C8—H8	110.2 (8)
C5—C6—C7	122.87 (11)	C9—C8—H8	107.6 (8)
C1—C6—C7	117.86 (11)	N1—C8—H7	110.2 (9)
O1—C7—N1	119.70 (11)	C9—C8—H7	109.0 (9)
O1—C7—C6	121.79 (11)	H8—C8—H7	107.6 (12)
N1—C7—C6	118.50 (10)	N2—C9—C8	179.61 (18)
C4—C5—C6	119.72 (13)	C3—C2—C1	119.98 (14)
C4—C5—H5	120.3 (9)	C3—C2—H2	120.6 (10)
C6—C5—H5	119.9 (9)	C1—C2—H2	119.4 (10)
C3—C4—C5	120.40 (14)	C4—C3—C2	120.20 (14)
C3—C4—H4	122.4 (10)	C4—C3—H3	121.1 (10)
C5—C4—H4	117.2 (10)	C2—C3—H3	118.7 (9)
C2—C1—C6	120.49 (13)	C7—N1—C8	120.25 (11)
C2—C1—H1	121.8 (9)	C7—N1—H6	121.2 (11)
C6—C1—H1	117.7 (9)	C8—N1—H6	118.2 (11)
N1—C8—C9	112.10 (11)		

### Hydrogen-bond geometry (Å, °)

**Table:**

*D*—H···*A*	*D*—H	H···*A*	*D*···*A*	*D*—H···*A*
N1—H6···O1^i^	0.819 (17)	2.021 (18)	2.8313 (14)	169

Symmetry codes: (i) *x*+1/2, *y*, −*z*+1/2.
